# A new perspective on the relationship between body and mind in the unconscious: The comparison between Freud and Merleau-Ponty

**DOI:** 10.3389/fpsyg.2023.1073362

**Published:** 2023-01-25

**Authors:** Lei Zhang, Xiaoli Yuan, Hanying Cui

**Affiliations:** ^1^School of Early-Childhood Education, NanJing XiaoZhuang University, Nanjing, China; ^2^Department of Psychiatry, Jingling Hospital, Nanjing, China; ^3^College of Marxism, Nanjing Agricultural University, Nanjing, China

**Keywords:** the unconscious, mind–body, topographical theory, psychoanalysis, body phenomenology

The unconscious is the core concept of psychoanalysis. Although both Freud and Merleau-Ponty used the unconscious to express their theories, each of them had their understanding of how the mind–body connection relates to the unconscious. For Freud, the unconscious derived from the libido of the human body participated in the construction of the mind. Thus, it is clear that Freud derived his concept of the unconscious based on mind–body duality. However, Merleau-Ponty believed that the unconscious itself was a way of being, which involved the interweaving of body and mind (Merleau-Ponty, 2005) or an “unreflected life, life as immediately experienced” (Phillips, 2017).

Freud used the term “unconscious” to refer to the repressed content which was not a part of consciousness. He then proposed that the unconscious is a dynamic and topographical system (Freud, 1915). Although Freud's definition of the unconscious became more complex, his theory mainly referred to two key questions: 1. What is the content of the unconscious? 2. How does the unconscious work? The answers to these questions are closely related to an understanding of the mind–body relationship of the unconscious. In this review, we discuss Merleau-Ponty's body phenomenological theory and propose a new interpretation of the mind–body relationship to the unconscious.

## 1. What is the content of the unconscious?

According to Freud, there are three different interpretations of the unconscious: the descriptive unconscious, the dynamic unconscious, and the structural unconscious. These interpretations suggest that the unconscious is all of the content outside the consciousness, including desire, childhood experiences, and unacceptable ideas and emotions (added in Freud's later theory). Furthermore, this definition includes two different ideas about the contents of the unconscious: (1) the ideas associated with the mind and (2) the feelings and desires associated with the body. Although the content of the unconscious discussed by Freud contained relevant elements of body and mind, it was regarded as two completely separate parts that require transformation (Carella, 1974; Nagel, 2016; Zhang et al., 2020). Freud continued his theoretical logic by using the unconscious as the boundary between body and mind, putting different contents into the unconscious to achieve his initial construction of using the unconscious as the bridge between body and mind. Freud (1915) suggested that “[the unconscious] goes beyond pure psychology and touches on the relations of the mental apparatus to anatomy.” Therefore, Freud regarded the unconscious as a mediator between mind and body, which suggests a duality of mind and body (Freud, 1961). Therefore, Iurato noted that “this binary logic is the essence of the unconscious” (Iurato, 2015).

Merleau-Ponty agreed with Freud's view on the content of the unconscious. Specifically, Merleau-Ponty believed that the content of the unconscious should be all of the content outside of consciousness, including desire, feelings, emotions, and some unconscious concepts (Brooke, 1986; Merleau-Ponty, 2010a). However, Merleau-Ponty did not try to divide these chaotic contents into two groups making the unconscious the boundary of body and mind. In other words, although the contents he proposes are similar to Freud's, they are neither physical nor mental.

In Merleau-Ponty's view, the content of the unconscious was mixed, experiential, and being itself. Furthermore, under the framework of Merleau-Ponty's theory, the unconscious was not a boundary or connection point, but the original state of the coexistence of the body and mind. Merleau-Ponty believed that the unconscious was more open and permeable, or an “echo of others in me, of me in others” (Merleau-Ponty, 2010b). Thus, Merleau-Ponty's view of the unconscious was more similar to the expression in the interweaving process of individuals and the world. He believed that the interwoven state could be described by his concept of the flesh (Merleau-Ponty, 1968). In short, the physical body is the common essence of the individual and the world, through which we, as subjects, can establish a specific relationship with the world (Mishara, 2012). In this relationship, the individual and the world are both composed of the flesh, and they wrap, perceive, and invade each other through the flesh. The content within psychological reach, such as ideas, is the visible part of the body, while meaning and desire are invisible. “The visible” refers to something that can be sensed: it is “a quality pregnant with a texture, the surface of a depth, a cross section upon a massive being, a grain or corpuscle borne by a wave of being” (Merleau-Ponty, 1968). “The invisible” refers to another dimension that cannot be sensed: it is the depth of the flesh and the source of the idea. Although the *visible* and *invisible* are two dimensions of the flesh, the visible is neither the deep structure nor the opposite of the invisible. Therefore, Merleau-Ponty may regard the relationship between the unconscious and conscious as visible and invisible in the body. The visible and invisible coexist and have a relationship of Ek-stasis which means the visible originates from the invisible. They are the two halves of the body. Therefore, the individuals' integration and understanding of the world do not occur in the resulting absolute consciousness, but in the interaction with the world. During this process, the body completes this connection as a homogeneous process with the world, “[t]he body proper embraces a philosophy of the flesh as the visibility of the invisible” (Merleau-Ponty, 1970). For example, prior studies have examined the guiding role of physical functions or symbols in the formation of the mind (Carignani, 2012; Austin and Sweller, 2017). Thus, the unconscious exists in the interweaving of the individual and the world, and the conscious is the expression of the meaning represented by the unconscious. Once this expression focuses on a certain part of the unconscious, it will lose its original meaning. The unconscious, as an invisible meaning, integrates the body and mind and is unified in being itself. Therefore, in the unconscious that Merleau-Ponty proposed, content is the whole experience of the subject. The experience cannot be distinguished between body and mind (Merleau-Ponty, 1970). It transcended the division of body and mind and existed in a fuzzier and more primitive way.

## 2. How does the unconscious work?

Freud described the organization and operation of the unconscious based on its dynamics and systematization. He regarded the unconscious as a part of his topographical theory, which enters consciousness by overcoming resistance. Therefore, the goal of psychoanalytic treatment was to turn the repressed unconscious content into conscious thought, which reflects Freud's topographical theories. In his first topographical theory, Freud proposed that the contents of the unconscious should be attached to the ideas to bypass the inspection mechanism before they can enter the consciousness. Similarly, in the second topographic theory, although Freud emphasized the unconscious was not only the repressed portion of the content but also the non-repressed part, he insisted that the unconscious and conscious are two parts that needed to be transformed. Additionally, in the second topographic theory, Freud constructed an unconscious–conscious hierarchical structure, that is, the unconscious and conscious were completely separated and suggested that the unconscious content should be attached to the ideas, and through its dynamic operation and repressive review enter a more idealized consciousness. Furthermore, Freud continued his view of the unconscious as the boundary between body and mind. From his view, the conscious contained more psychological characteristics, while the content of the unconscious required a series of dynamic operations of the brain before it could enter the conscious, get rid of the physical characteristics, and completely acquire its psychological characteristics (Kirsch, 2019). Freud's construction of the topographic theory was very delicate, but the basic assumption continues to be a binary relationship between body and mind in the unconscious. In this regard, Merleau-Ponty rejected the diametrically opposed structure between the unconscious and conscious. He believed that the unconscious and conscious were not a distinct upper and lower level of structure, but a relationship between focus and background. Specifically, he believed the unconscious and conscious were on the same level, similar to symbiotic relations.

First, the unconscious could not be acquired through conscious reflection. In Freud's view, the unconscious was *latent*, and it could only be revealed through the analysis of many phenomena in conscious life, including verbal errors, associations, dreams, and actions. Freud's original intention was to find an interface for the secondary structure of the unconscious–conscious, but he also showed that the unconscious was not a subordinate structure and that it existed in the process of life practice with the consciousness. Coincidentally, the main goal of current psychoanalysis is also to activate the unconscious physical and mental connection of patients, so that they can not only form explanations but also feel alive (Lombardi, 2018).

Second, the conscious itself is not purely psychological, it has physical characteristics. For example, Freud noted in *the unconscious* that consciousness also originates from the body. The mind that is centered on the conscious and the body are not binary opposites. Thus, the dynamic process within the unconscious cannot be regarded as a purely psychological monitoring and suppression system in the conscious, but rather as a display process in the interweaving process of body and mind. The former has traces of psychological aspects, while the latter is the common activity of body and mind, and a common operation between individuals and the world. In this regard, Merleau-Ponty also “elaborates his own non-Freudian “return to Freud,” and “consciousness and unconscious redefined in terms of body” (de Saint Aubert, 2017). The unconscious is the latent system in that the body constitutes a conscious through perception, behavior, and so forth, while the conscious, like the visible, is the manifestation of this system. The unconscious and conscious are interwoven and coexist. Furthermore, the relationship between the unconscious and conscious is no longer a dynamic operation under the topography of the mind, but the unconscious encompasses the conscious, which is the potential and anonymous basis of individual interpersonal relationships. Therefore, the conscious cannot determine which unconscious enters through the monitoring organization, but the conscious itself expresses the unconscious.

In Merleau-Ponty's view, the dynamic structure of the unconscious includes the characteristics of desire, whether it promotes ideas and behaviors or suppresses forces. The desires existed in the body but were also entangled in psychological activities. However, from Freud's perspective, the libido in the unconscious includes somatic sources (Freud, 1938; Moya and Larrain, 2016) and faces the world (Mazis, 2016). For Merleau-Ponty, the representative force of the unconscious libido was no longer a simple sexual desire. The polymorphism and promiscuity were not only the attributes of infantile sexuality but also the attributes of being itself (Phillips, 2017). Therefore, exploration of the unconscious is not only accomplished through “talking cure,” but also through more non-verbal methods. For example, Freud's successors focused more on non-verbal communication methods than Freud (de Peyer, 2016; Zhang et al., 2019). Although Freud hinted that the content of the unconscious was the integration of body and mind, he also focused more on the analysis of the unconscious and non-verbal factors through “talking cure.” For instance, when treating Emmy, he described the facial expressions and intonations in detail (Breuer and Freud, 1895). Freud's successors have found that the talking cure cannot cover the unconscious, but it may help facilitate understanding of the unconscious through non-verbal clues. Such non-verbal communication is often regarded as an unconscious process. Merleau-Ponty noted that this process may be established and effective due to the indistinguishability and interweaving of body and mind in the unconscious framework. Furthermore, Merleau-Ponty described a method of communication between patients and psychoanalysts based on the existence of the individual itself, which is often regarded as transference.

Transference is the mutual opening and interweaving between the subject and the existence of the subject, which is the best expression of the dynamic structure of the unconscious. In classic psychoanalysis, Freud believed that transference would harm the psychoanalysts' analysis of the patients unconscious because of the duality of body and mind in the unconscious. However, after exploring the role of transference and counter-transference in understanding the unconscious processes interwoven between psychoanalysts and patients, some of Freud's successors believed that transference and counter-transference could not only activate the psychological connection between the psychoanalyst and patient but also improve the emotional connection between them and promote their profound understanding of the unconscious. Indeed, this understanding is not only conceptual but also contains the non-verbal part of the patients (Birksted-Breen, 2019). Merleau-Ponty (1988) wrote: “The psychological mechanisms of introduction and projection, instead of appearing as mental operations, should be understood as the very modalities of the activity of the body.”

Merleau-Ponty's belief provides a different perspective from Freud's theory of the interweaving of body and mind to explain the content and operating characteristics of the unconscious. From the perspective of Merleau-Ponty's theory, the relationship between body and mind is intertwined in the unconscious. Therefore, the concepts of desire and psychological characteristics with their physical origin in the unconscious were both visible and invisible within the same body. The psychological structure formed by the unconscious and conscious was the relationship between background and focus, not the hierarchical structure. This indicates that the unconscious is neither a part of Husserl and Fleischer (1966)'s discussion of consciousness nor an abstract representation manipulation under the perspective of disembodied cognition (Anderson, 2003), but can be considered as embodied cognition, which is unrepresented and unformalized in the background of the world.

## Author contributions

LZ and HC contributed to the conception of the study. LZ and XY wrote the manuscript. All authors contributed to the article and approved the submitted version.
